# Insecticide resistance status of malaria vectors *Anopheles gambiae* (*s.l.*) of southwest Burkina Faso and residual efficacy of indoor residual spraying with microencapsulated pirimiphos-methyl insecticide

**DOI:** 10.1186/s13071-020-04563-8

**Published:** 2021-01-18

**Authors:** Dieudonné Diloma Soma, Barnabas Zogo, Domonbabele François de Sales Hien, Aristide Sawdetuo Hien, Didier Alexandre Kaboré, Mahamadi Kientega, Anicet Georges Ouédraogo, Cédric Pennetier, Alphonsine Amanan Koffi, Nicolas Moiroux, Roch Kounbobr Dabiré

**Affiliations:** 1 Institut de Recherche en Sciences de la Santé, Bobo-Dioulasso, Burkina Faso; 2 Université Nazi Boni, BP 109, Bobo-Dioulasso, Burkina Faso; 3 MIVEGEC, IRD, CNRS, Univ. Montpellier, Montpellier, France; 4 Institut Pierre Richet, Institut National de Santé Publique, Bouaké, Côte d’Ivoire

**Keywords:** Vector control, Resistance, Chemical analysis, Burkina Faso, IRS

## Abstract

**Background:**

The rapid spread of insecticide resistance in malaria vectors and the rebound in malaria cases observed recently in some endemic areas underscore the urgent need to evaluate and deploy new effective control interventions. A randomized control trial (RCT) was conducted with the aim to investigate the benefit of deploying complementary strategies, including indoor residual spraying (IRS) with pirimiphos-methyl in addition to long-lasting insecticidal nets (LLINs) in Diébougou, southwest Burkina Faso.

**Methods:**

We measured the susceptibility of the *Anopheles gambiae* (*s.l.*) population from Diébougou to conventional insecticides. We further monitored the efficacy and residual activity of pirimiphos-methyl on both cement and mud walls using a laboratory susceptible strain (Kisumu) and the local *An. gambiae* (*s.l.*) population.

**Results:**

*An. gambiae* (*s.l.*) from Diébougou was resistant to DDT, pyrethroids (deltamethrin, permethrin and alphacypermethrin) and bendiocarb but showed susceptibility to organophosphates (pirimiphos-methyl and chlorpyrimiphos-methyl). A mixed-effect generalized linear model predicted that pirimiphos-methyl applied on cement or mud walls was effective for 210 days against the laboratory susceptible strain and 247 days against the local population. The residual efficacy of pirimiphos-methyl against the local population on walls made of mud was similar to that of cement (OR = 0.792, [0.55–1.12], Tukey’s test *p*-value = 0.19).

**Conclusions:**

If data on malaria transmission and malaria cases (as measured trough the RCT) are consistent with data on residual activity of pirimiphos-methyl regardless of the type of wall, one round of IRS with pirimiphos-methyl would have the potential to control malaria in a context of multi-resistant *An. gambiae* (*s.l.*) for at least 7 months.
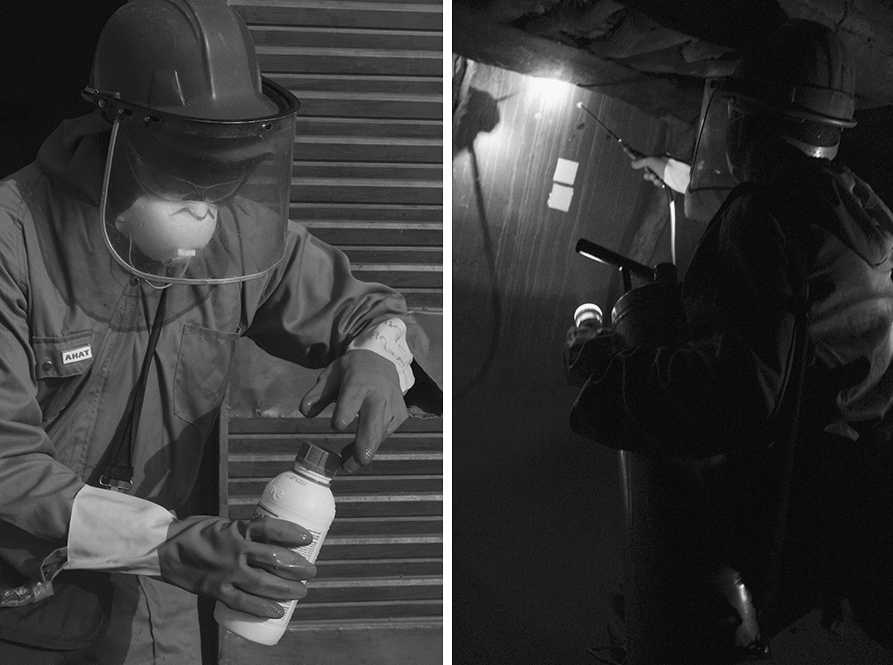

## Background

Long Lasting Insecticidal mosquito Nets (LLINs) and indoor residual spraying (IRS) are major malaria vector control strategies [1]. Both strategies have had substantial impacts on the malaria burden over the past 15 years. Indeed, LLIN and IRS accounted for an estimated 68 and 11% of the malaria averted cases, respectively, between 2000 and 2015 [2]. Historically, IRS based on DDT was the cornerstone of the global malaria eradication campaign that led to the elimination of malaria in 15 countries in Europe and America during the 1950s and 1960s. In Africa, however, these campaigns were not widely implemented because of a number of reasons including limited resources [3]. Subsequently, the coverage of IRS has dropped considerably in favor of LLINs. Until 2014, very few African countries still considered IRS as a prior action in malaria vector control [4]. More recently in 2017, IRS was implemented, either alone or in combination with LLINs, in 40 African countries [5]. Interest in combining IRS with LLIN seems to have increased in recent years across Africa because of the raise of pyrethroids resistance within the main major malaria vectors [6]. As of 2017, the arsenal of insecticides recommended for IRS has been improved considerably, making available five classes of insecticides including organochlorines, carbamates, organophosphates, pyrethroids and neonicotinoids [7, 8]. The Global Plan for Insecticide Resistance Management (GPIRM) recommends rotation of non-pyrethroid insecticides for IRS in areas where IRS and LLIN are combined [9]. However, options available for continued insecticide rotation are very limited in many endemic countries because resistance to multiple insecticide classes is very common in vector populations. According to WHO, resistance to organochlorines and carbamates was confirmed, respectively, in 62.4% and 30.6% of the sites tested in Africa between 2010 and 2016 [6]. Resistance to organophosphate was less common, with 14.1% of the sites tested in Africa confirming its occurrence [6].

In addition to the insecticide physiological resistance, a variety of factors can affect the effectiveness of IRS. Indeed, the residual life and efficacy of the insecticide used can vary according to the formulation, the quality of sprays and the type of walls (cements, mud, wood) [10, 11].

This study was part of a randomized-controlled trial (RCT) in the rural area of Diébougou, Southwest Burkina Faso, aiming at investigating whether the use of complementary strategies together with LLINs affords additional reduction in malaria transmission and cases. One of the strategies evaluated was IRS with microencapsulated formulation of pirimiphos-methyl. Microencapsulation is a technology that allows insecticides to last longer on substrates than usual [12]. In the present study, we tested the susceptibility of the *An. gambiae* (*s.l.*) population from the rural area of Diébougou (southwest Burkina Faso) to conventional insecticides (including those of LLINs and IRS used in the RCT). Furthermore, we assessed the residual bio-efficacy of pirimiphos-methyl on mud and cement walls treated during the trial using a susceptible strain of *Anopheles gambiae* (*s.s.*) (*Kisumu*) and a wild *An. gambiae* (*s.l.*) population.

## Methods

### Study area

**Fig. 1:**
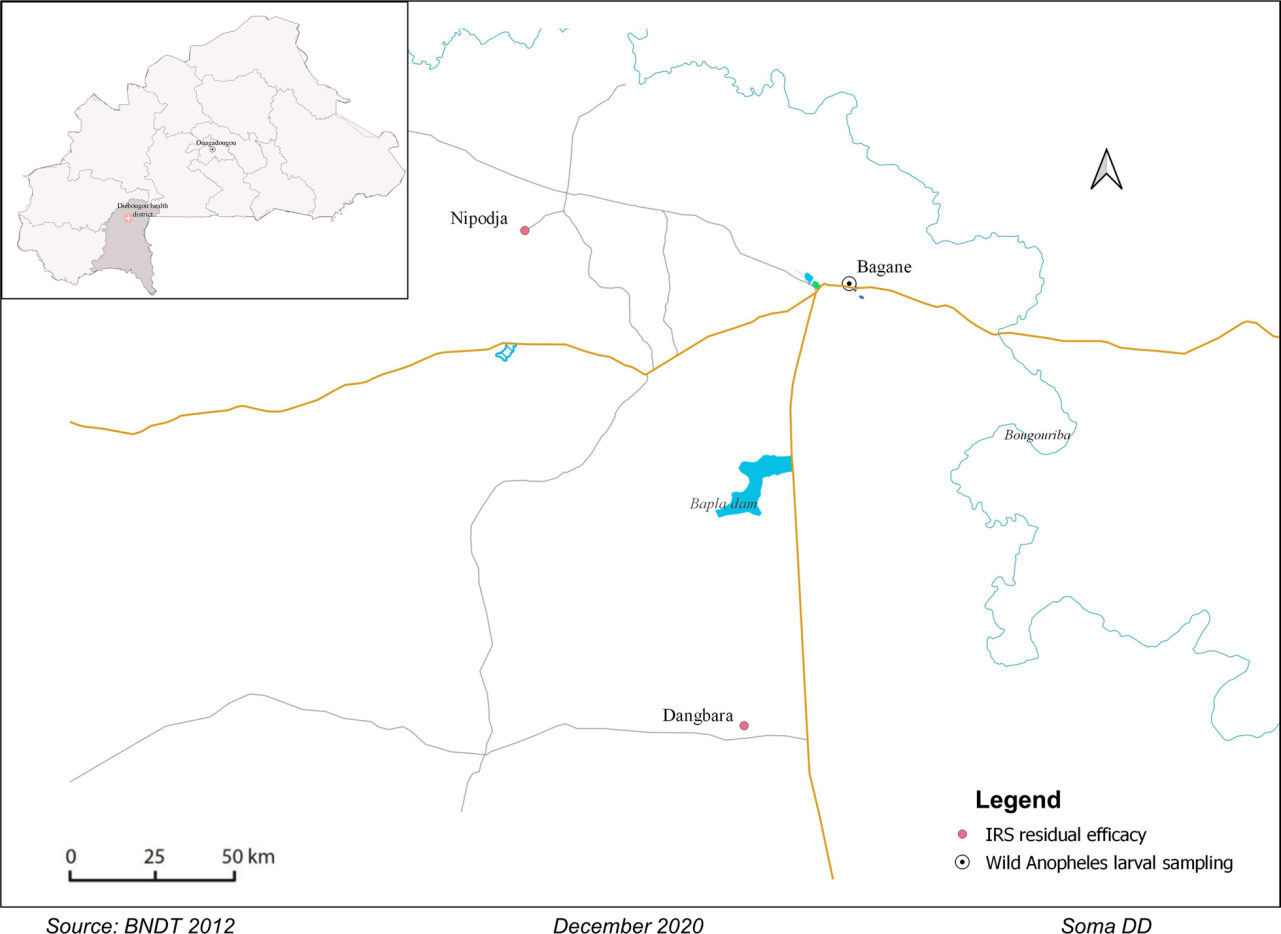
Location of the study areas

This study was carried out in two villages, Dangbara (− 3.284°; 10.766°) and Nipodja (− 3.383°; 10.988°), located in the Diébougou health district in southwest Burkina Faso (Fig. 1). These villages were selected (based on their accessibility and the presence of both banco- and cement-made houses) among the five villages which received a pirimiphos-methyl IRS intervention in a randomized control trial run in Diébougou, southwest Burkina Faso [13]. The Diébougou area is characterized by an average annual rainfall of 1200 mm. The climate is tropical with two seasons: one dry season from October to May and one rainy season from June to September. Average daily temperature amplitudes are 18–36 °C, 25–39 °C and 23–33 °C in the dry cold (November to February), dry hot (March to May) and rainy season (June to October), respectively. Agriculture is the main economic activity in the area, followed by artisanal gold mining and coal and wood productions [14, 15].

### House spraying

Actellic^®^300CS (Syngenta AG, Basel, Switzerland) was applied at a target dosage of 1 g of active ingredient (pirimiphos-methyl) per square meter (1g a.i./m^2^) in all houses of both villages in September 2017. We performed IRS using Hudson^®^ X-pert spray pumps (H.D. Hudson Manufacturing Co., Chicago, IL). The spray pumps (15 l) were fitted with a 1.5 bar control flow valve on the lance pressure and equipped with a ceramic 8002E nozzle according to the WHO guidelines [16]. The spraying was performed by volunteers from the local communities who were trained by the National Malaria Control Program (NMCP) staff on a previous IRS campaign in Diébougou in 2012. We re-trained the spray operators and supervisors prior to IRS operations in the villages.

### Safety precautions

We took standard safety precautions with regard to mixing, handling and spraying insecticides [16, 17]. Spray operators and supervisors used appropriate protective equipment (gloves, hats, overalls, boots and facemasks). Spray operators, supervisors and householders were provided with an illustrated information sheet on the study, the possible adverse events in case of inappropriate spraying and safety precautions. We properly disposed of the leftover insecticides and bottles according to standard procedures [16]. The householders were advised by IRS operation teams about safety precautions to avoid possible risks during and after spraying. They were advised to remain outside the rooms during spraying and until 3 h after spraying. Adult heads of households were advised to ask their children not to intentionally touch the sprayed walls for at least 1 day after spraying, as the walls remained wet for about 1 day. We advised the householders, if possible, to not scrub, mutilate or plaster the walls until the end of the study. The medical team of the Diébougou health district participated in the trial to attend to any medical illnesses of the inhabitants or IRS operation team members.

### Chemical analysis

Before spraying, we attached Whatman No. 1 filter papers (10 × 10 cm) to the four inner walls of six randomly selected houses (three made of mud walls and three made of concrete walls) per village. On each four inner walls, two types of filter papers (one plasticized and one classical) were fixed to test for a possible migration of the insecticide from the filter papers to the wall as hypothesized by Moiroux et al. [18]. Plasticized and classical papers were fixed in areas where spray overlap was unlikely to ensure that the quantity of insecticide was constant. We also marked positions of filter papers on the wall to avoid carrying out subsequent cone bioassays at such surfaces. The filter papers were removed 24 h after spraying and placed individually in aluminum foil with appropriate labels (village code, house number, type of surface and date of spraying). We stored the packed samples in a refrigerator at + 4 °C before sending them to the WHO collaborating center, Gembloux, Belgium, for analysis of the pirimiphos-methyl content.

### Insecticide resistance of wild *An. gambiae* (*s.l.*) and residual efficacy

Both wild *An. gambiae* (*s.l.*) from the study area and the susceptible *An. gambiae* (*s.s.*) Kisumu strain (KISUMU1, MRA-762, VectorBase stable ID VBS0000026 on vectorbase.org) were used in the following bioassay. We collected *Anopheles* sp. larvae in Bagane (3.150°; 10.575°, unsprayed village). Larvae were reared in the insectary of IRSS (temperature 27 ± 2 °C; relative humidity: 70 ± 5%; 12 h:12 h light:dark regimen) to adulthood. We fed larvae every day with Tetramin^®^ baby fish food. After emergence, mosquitoes were identified to species level using morphological keys [19]. Adult *Anopheles* mosquitoes belonging to *An*. *gambiae* (*s.l.*) were provided with a sugar solution (10%), until their use for bioassays.

We tested the susceptibility of an *An*. *gambiae* (*s.l.*) population from Bagane (F0 derived from larval collections) to six insecticides using the standard WHO protocol [20]. We exposed four replicate samples of 20–25 non-blood-fed females, 3–5 days old, *An*. *gambiae* (*s.l.*), for 60 min to each insecticide. We recorded mortality after 24 h. Four insecticide classes were tested: carbamates (bendiocarb 0.1%), pyrethroids (alphacypermethrin 0.05%, permethrin 0.75% and deltamethrin 0.05%), organochlorine (DDT 4%) and organophosphates (chlorpyrifos-methyl 0.4% and pirimiphos-methyl 0.25%) [20]. As a negative control, two replicates of the same batch of mosquitoes were exposed to silicon oil-impregnated papers. As a positive control, four replicates of susceptible *An. gambiae* (*s.s.*) Kisumu mosquitoes were tested with all insecticides.

In the same 12 houses randomly selected for chemical analysis, WHO cone tests were performed on days 2, 30, 60, 90, 120, 150, 180 and 210 post-spraying using both susceptible *An. gambiae* (*s.s.*) and wild *An*. *gambiae* (*s.l.*). Bioassays were further performed on day 360, but only using the susceptible *An. gambiae* (*s.s.*) Kisumu. In each house, we performed WHO cone tests on the four inner walls according to WHO guidelines [20]. A WHO cone test consists of introducing 10 to 15 unfed mosquitoes (3–5 days old) into a standard WHO cone for 30 min of exposure to the wall. As a control, four cone tests were performed on four unsprayed blocks. After exposure time, mosquitoes were placed in 150 ml plastic cups (1 replicate per cup) with 10% sucrose solution. All mosquitoes were held for 24 h in the laboratory (27 °C ± 2 °C and 70% ± 5% relative humidity) to assess mortality.

### Statistical analysis

We analyzed insecticide susceptibility of the wild *An. gambiae* (*s.l.*) using a binomial generalized model with the mortality recorded in each tube as the response and the insecticide as fixed effect. The ‘brglm’ function of the ‘brglm’ package [21] in the software ‘R’ [22] was used for this analysis. It allows to fit binomial-response regression models using the bias-reduction method developed by Firth [23]. These procedures return estimates with improved frequentist properties (bias, mean squared error) that are always finite even in cases where the maximum likelihood estimates are infinite (data separation). We used the ‘emmeans’ function of the ‘emmeans’ package to calculate estimated marginal means (EMM) of mortality for each insecticide and 95% confidence intervals [24].

We compared pirimiphos-methyl concentrations on filter papers using a linear (Gaussian) mixed effect model (LMM) with the wall surface (mud or cement), type of filter paper (classical or plasticized) and interaction as fixed effects. The house and the wall in the house were set as nested random intercept. Tukey’s post-hoc method was used to do multiple comparisons among modalities of the fixed terms (wall surface and paper type) using the ‘emmeans’ function of the ‘emmeans’ package [24]. Mean differences (MD) and their 95% confidence interval were calculated.

For each strain, we analyzed the mortality rate recorded in cone bioassays using a binomial response mixed effect model. We set the wall surface (cement or mud), time after spraying (log-transformed) and interactions as fixed effects. The house was set as a random intercept. Odds ratio (OR) and their 95% confidence intervals (CI) were computed. The ‘predict’ function in R applied on the bioassay mortality models was used to predict the time at which mortality fell under the 80% mortality threshold. We computed 95% confidence intervals of predictions.

## Results

### Insecticide susceptibility bioassays

**Fig. 2:**
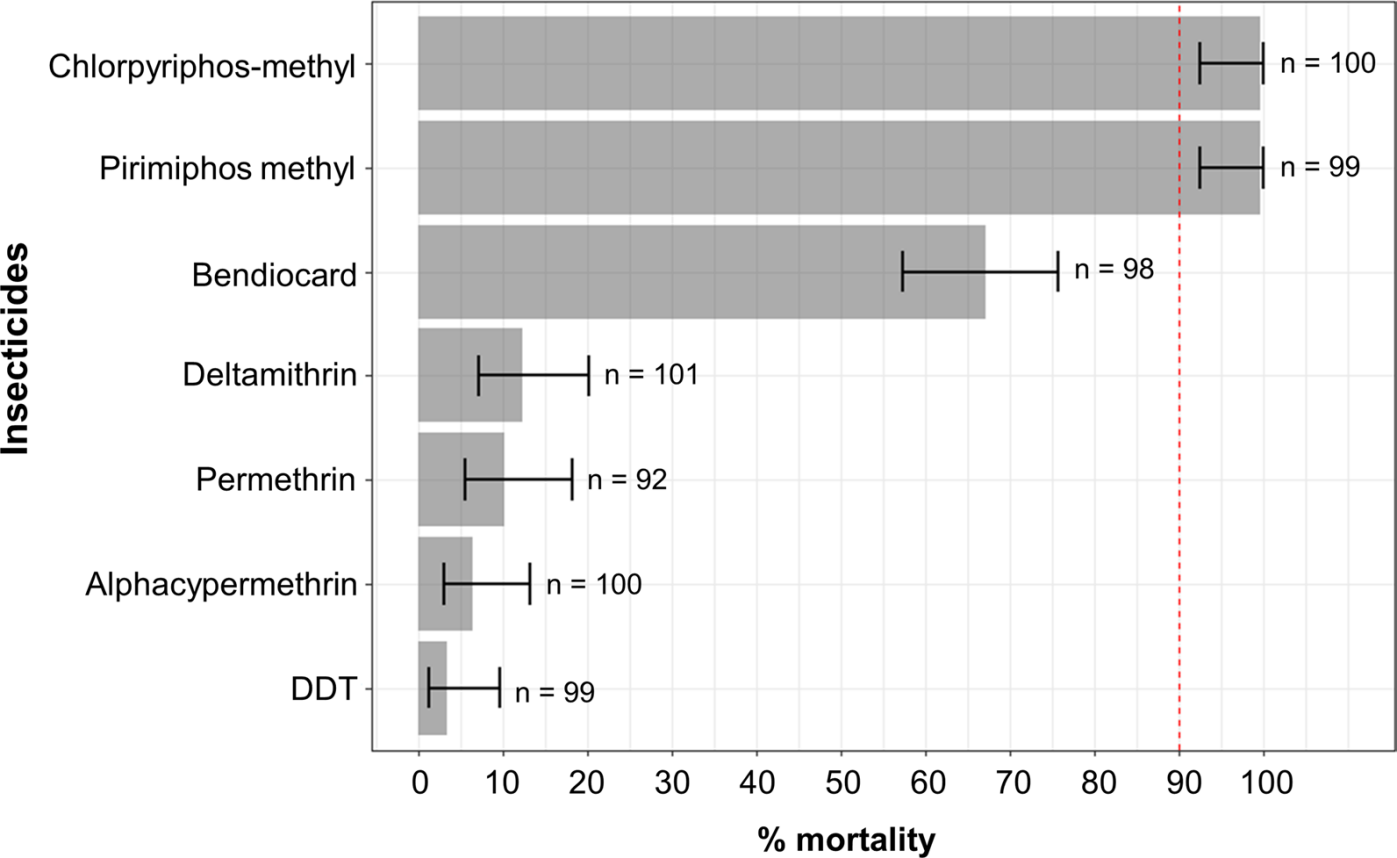
Susceptibility of wild *An. gambiae* (*s.l.*) from southwest Burkina Faso to seven insecticides used for malaria control. Bars indicate estimated marginal means (EMM) of mortality as predicted by a generalized linear model. Error bars represent the 95% confidence intervals of the EMMs. If mortality falls under 90% (red dashed line), the mosquito population is considered resistant to the tested insecticide

*The An. gambiae* (*s.l.*) population from Diébougou was highly resistant to DDT and pyrethroids (alphacypermethrin, permethrin and deltamethrin), with mortality rates < 15%, as recorded in WHO susceptibility bio-assays (Fig. 2). This population was also resistant to bendiocarb (mortality rate = 67%, 95% CI [57; 75]). However, it was fully susceptible (100% mortality) to both organophosphate insecticides tested (pirimiphos-methyl and chlorpyriphos-methyl). No mortality (0%) was observed in the negative control tubes (silicon oil). Mortality rate of the susceptible *An. gambiae* (*s.s.*) Kisumu mosquitoes for all the insecticides tested was 100%.

### Chemical analysis

**Fig. 3:**
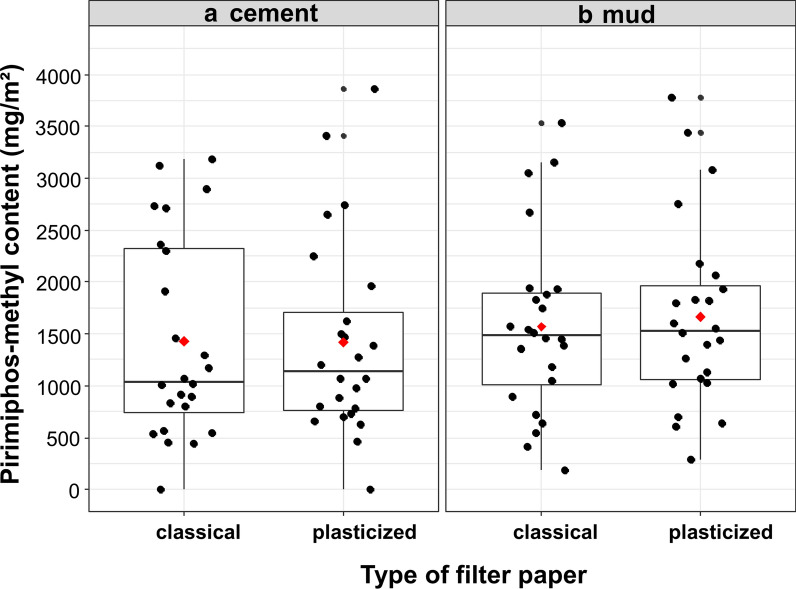
Applied doses of pirimiphos-methyl on cement (**a**) and mud (**b**) walls. Red diamonds show the mean concentrations of pirimiphos-methyl on filter papers. Boxes show first and third quartiles as well as the median concentration. The whiskers extend to the largest and lowest values that are no further than 1.5 * IQR (where IQR is the interquartile range or distance between the first and third quartiles). Black dots represent concentration measured for all filter papers

On cement walls, chemical analysis indicated that the mean concentrations of pirimiphos-methyl on classical and plasticized filter papers were 1428 mg/m^2^ (95% CI [719; 2136]) and 1421 mg/m^2^ (95% CI [713; 2130]), respectively (Fig. 3a). We did not find a difference in pirimiphos-methyl concentration between the classical and plasticized papers applied on cement walls: mean difference (MD) = 6.13 mg/m^2^ (95% CI [− 309; 322]), Tukey’s test *p*-value = 0.96.

On mud walls, chemical analysis indicates that the mean concentrations of pirimiphos-methyl on classical and plasticized papers from sprayed houses were 1569 mg/m^2^ (95% CI [861; 2278]) and 1665 mg/m^2^ (95% CI [957; 2373]), respectively (Fig. 3b). We were not able to find a difference in pirimiphos-methyl concentration between classical and plasticized papers applied on mud walls (MD = − 95.76, 95% CI [− 411; 220], Tukey’s test *p*-value = 0.54). Moreover, the pirimiphos-methyl concentration on papers placed on cement did not differ from that on mud walls (MD = − 193, 95% CI [− 1039; 653], Tukey’s test *p*-value = 0.65).

### Insecticide residual efficacy

**Fig. 4:**
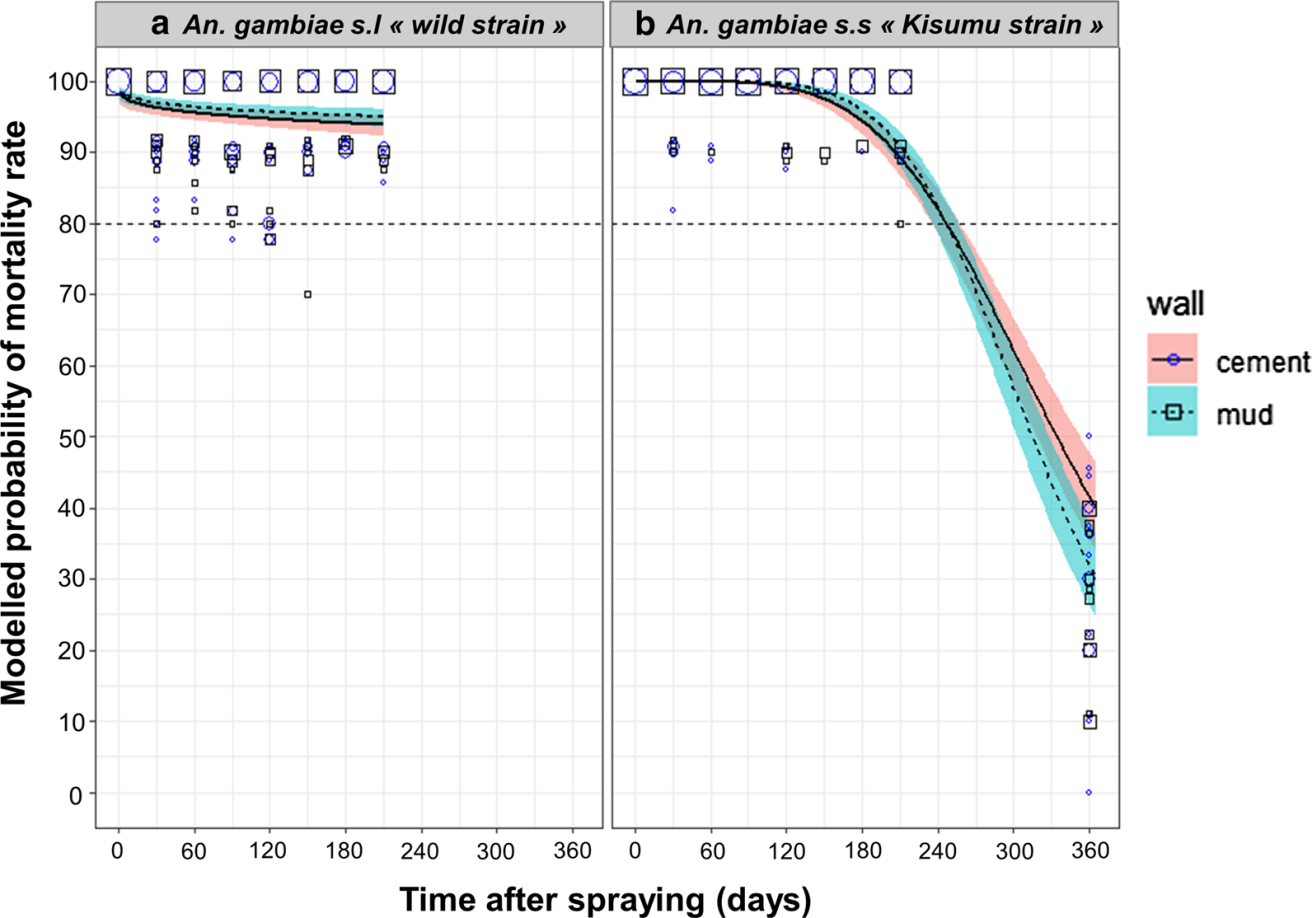
Efficacy (mortality) over time of indoor residual spraying of pirimiphos-methyl against wild *An. gambiae* (*s.l.*) (**a**) and susceptible *An. gambiae* s.s (**b**). Mortality rates were predicted from a binomial-response mixed effect model. Pirimiphos-methyl at 4 ml/m^2^ targeted dose applied on mud (dashed lines) or cement (solid lines) walls are compared. Gray areas are 95% confidence interval of predicted means. Mortality values measured on the field are shown as blue circles (cement) and black squares (mud) of size proportional to the number of values (maximum = 20)

Predictions from the mortality model of *An. gambiae* (*s.l.*) wild strain showed that pirimiphos-methyl efficacy remained > 80% until the last test (i.e. 210 days after spraying) on both cement and mud walls (Fig. 4a). We were not able to find a difference in residual efficacy of pirimiphos-methyl between cement and mud walls with *An. gambiae* (*s.l.*) wild strain (OR = 0.792, 95% CI [0.55; 1.12], *p*-value = 0.19).

With the susceptible *An. gambiae* (*s.s.*)* Kisumu* strain, for which a supplementary test was done on day 360 post-spraying, predictions from the mortality model show that pirimiphos-methyl treatment was effective (mortality > 80%) until the 247th day post-spraying, on both cement and mud walls (Fig. 4b). The residual efficacy of pirimiphos-methyl was lower on mud than on cement walls (OR = 0.257, 95% CI [0.07; 0.86], *p*-value = 0.02).

## Discussion

Insecticide resistance management has remained a major challenge for malaria control and elimination for years [6, 9]. This is, in large part, because malaria vectors are developing resistance to most of the insecticides currently used in public health [6]. Insecticide susceptibility assays showed high resistance of *An. gambiae* (*s.l.*) from Diébougou to all pyrethroids tested (deltamethrin, permethrin and alphacypermethrin). Our results are consistent with that of a recent investigation conducted in an area of southwest Burkina Faso in 2016 [25]. However, compared with data collected pre-2010, this study suggests that the prevalence of pyrethroid resistance has increased considerably over time [26–28]. In addition, DDT and Bendiocarb induced respectively 4% and 67% mortality rates, indicating a multi-resistance of the wild *An. gambiae* (*s.l.*) populations to pyrethroids, organochlorines and carbamates.

Many mechanisms might be involved in this multiple resistance. Indeed, both L1014F and L1014S *kdr* mutations that confer cross-resistance to organochlorine and pyrethroids were recorded in a neighboring population from Diébougou [25]. The same authors also describe the presence at low frequency of the *ace-1* mutation that confers cross-resistance to both carbamates and organophosphorous and evidenced the presence of metabolic resistance mechanisms (esterase and GST) that may confer resistance to all tested families of insecticides [6]. The large-scale use of LLINs across the country [29] might have contributed to the selection of these resistance mechanisms, particularly those involved in pyrethroid resistance, as well as the intense use of insecticides in agriculture [30–32]. Nevertheless, the wild population of *An. gambiae* (*s.l.*) from our study area was found to be fully susceptible to organophosphorus compounds (chlorpyrifos-methyl and pirimiphos-methyl). These data were strengthened by the results of the WHO cone bioassay done in houses of two villages sprayed with pirimiphos-methyl CS. Indeed, the duration of residual efficacy (mortality > 80%) of pirimiphos-methyl IRS on mud and cement walls was > 7 months against wild strains of *An. gambiae*. Unfortunately, we were not able to determine the precise effective duration because further testing was not performed beyond 7 months.

The mortality model predicted that the residual efficacy of pirimiphos-methyl IRS lasted for 247 days (8–9 months) against the susceptible *An. gambiae* Kisumu strain. In Benin, pirimiphos-methyl sprayed in experimental huts has shown 9 and 6 months of effective residual efficacy on cement and mud substrates, respectively, against susceptible *An. gambiae* Kisumu [33]. However, these durations dropped to 5 months on both substrates in houses of northern Benin with susceptible *An. gambiae* Kisumu [34]. In Ivory Coast, 5 and 7 months of residual efficacy were observed on mud and cement walls, respectively, against *An. gambiae* Kisumu [35]. In Ethiopia, Yewhalaw and colleagues observed a 6-month residual efficacy against a susceptible strain of *An. arabiensis* on mud substrates [36]. In Tanzania, pirimiphos-methyl displayed 3 to 6 months of residual efficacy depending on the substrate [37]. In a multi-country study [38], pirimiphos-methyl CS duration of residual efficacy ranged from 2 to 9 months. Many factors such as quality of spraying [39], vector resistance [6, 40], season/climate [41] and wall modifications post-application [42] can explain the observed differences in residual efficacy between sites and studies.

In this study, we did not find any difference in the residual efficacy between cement and mud surfaces. However, according to the results of the above-mentioned studies, insecticides often performed poorly on mud surfaces, probably because these surfaces are porous and hence absorb a quantity of the applied insecticide. Our results suggest that mud walls in the rural area of Diébougou might be less porous. The absence of difference in the residual efficacy between cement and mud surfaces could also be explained by the quantity of insecticides sprayed on the walls, which exceeds the recommended dose. Indeed, chemical analysis of classical filter papers showed that applied target dose ratios were 1.43 [0.81; 2.05] on cement surfaces and 1.57 [0.95; 2.19] on mud surfaces. This would indicate that the residual efficacy of pirimiphos-methyl CS would be reduced if the applied dose was within the recommended ± 25% limit of the target dose. In this study, we have tested two filter papers: classical and plasticized papers. Plasticized papers were tested because Moiroux et al. [18] found that the concentration of alpha-cypermethrin on filter paper placed on mud walls was lower than on filter papers placed on cement walls. They hypothesized that a quantity of the insecticide applied on mud surfaces may migrate from the filter papers to the wall, diminishing the insecticide content on classical papers placed on mud surfaces. Consequently, they suggest the use of plasticized papers to address the issue [18]. Here, we did not find any differences in concentration between papers placed on mud or cement surfaces. Further replications of this experiment are nevertheless recommended as we did not use the same insecticide as Moiroux et al.

To date, only microencapsulated formulation of pirimiphos-methyl and new formulations of clothianidin (neonicotinoids) (SumiShield^®^ 50WG and Fludora Fusion^®^ WP-SB) have the potential to be effective for > 6 months [4, 7]. This was confirmed in this study for pirimiphos-methyl. For clothianidin, no field trial was carried out to evaluate its residual efficacy as IRS in southwest Burkina Faso but local vector populations were shown to be susceptible [43]. Therefore, pirimiphos-methyl and clothianidin used in rotation or mosaic might constitute an effective insecticide resistance management strategy in southwest Burkina Faso.

## Conclusions

The *Anopheles gambiae* (*s.l.*) population from the rural area of Diébougou, in the southwest of Burkina Faso, was resistant to DDT, all pyrethroids tested and bendiocarb. In contrast, the same population was susceptible to both OPs tested (pirimiphos-methyl and chlorpyrimiphos-methyl). This result was further supported by the residual efficacy of pirimiphos-methyl IRS, which lasted > 7 months on cement and mud walls against both susceptible *An. gambiae* (*s.s.*) Kisumu strain and wild *An. gambiae* (*s.l.*) populations. If data on malaria transmission and malaria cases (as measured trough the RCT) are consistent with data on residual activity of pirimiphos-methyl regardless of the type of wall, one round of IRS with pirimiphos-methyl would have the potential to control malaria in a context of multi-resistant *An. gambiae* (*s.l.*) for at least 7 months.

## Data Availability

All relevant data are within the manuscript and its Additional files. The datasets used during this article are fully available without restriction.

## Abbreviations

IRS: Indoor residual spraying
LLIN: Long-lasting insecticidal net
WHO: World Health Organization
GPIRM: Global Plan for Insecticide Resistance Management
INSD: Institut National de la Statistique et de la Démographie
NMCP: National Malaria Control Program
IRSS: Institut de Recherche en Sciences de la Santé
